# Factors Influencing Induction of Labor Success in Riyadh, Saudi Arabia

**DOI:** 10.1155/2024/1637424

**Published:** 2024-09-26

**Authors:** Renad A. Alshalan, Rwan K. Alarfaj, Yazeed A. Almojel, Yara AlHaddad, Rahaf Alsomali, Maysoon Al Adham

**Affiliations:** ^1^ College of Medicine King Saud Bin Abdulaziz University for Health Sciences, Riyadh, Saudi Arabia; ^2^ King Abdulaziz Medical City, Riyadh, Saudi Arabia

## Abstract

**Introduction:**

The physiological process by which the fetus and placenta are delivered from the uterus and pass through the vaginal canal for delivery is known as labor. Induction of labor involves deliberately initiating labor before it occurs naturally, using medical interventions or techniques to stimulate contractions and initiate the birthing process.

**Aim:**

This study aimed to investigate the factors that influence the success of labor induction procedures in Riyadh, Saudi Arabia, from January to April 2023. *Subject and Methods*. This retrospective chart review was conducted at the National Guard Hospital in Riyadh, Saudi Arabia. Data were collected from the patient chart of those who underwent labor induction from January to April 2023. The collected data were tabulated and cleaned in MS Excel. Final data were transferred to SPSS for subsequent data analysis.

**Results:**

Five hundred and thirty-one pregnant women were analyzed. 52.7% were aged 30 years or below. The most common indication of IOL was post-dated pregnancy (26.2%). 62% were normal deliveries, indicative of IOL success, while 31.1% were cesarean deliveries, indicative of IOL failure. In univariate analysis, women with lower gravidity (≤3) and who had received Propess were associated with cesarean delivery. In a multivariate regression analysis, women who received Prostin and increased parity were identified as the significant independent predictors of IOL success.

**Conclusion:**

IOL's success was dependent primarily on increasing parity and Prostin administration. However, lower gravidity (≤3) and Propess medication could lead to operative procedures among pregnant women. Hence, it is necessary to carefully assess the condition of pregnant women before directing them to IOL.

## 1. Introduction

The physiological process by which the fetus and placenta are delivered from the uterus and pass through the vaginal canal for delivery is known as labor [1]. Induction of labor (IOL) involves the deliberate initiation of labor before it occurs naturally [2]. Bishop score can be used to assess the cervix favorability for labor induction [3]. IOL may be achieved by a range of approaches, including both pharmacological and nonpharmacological procedures [4]. Various techniques, including the use of cervical ripening agents, artificial rupture of membranes, and uterine stimulation with oxytocin or prostaglandins, may be used to induce labor, with prostaglandin preparations commonly employed when the cervix is not favorable [4–6]. Extensive global literature has explored factors influencing the success or failure of labor induction. For example, a critical analysis conducted at the University of Newfoundland in Canada highlighted the importance of maternal factors like height and BMI in induction success, with younger, taller, and lower BMI women having higher success rates and vaginal delivery within 24 hours. Fetal factors such as birth weight over 3.5 kg and advanced gestational age increase the likelihood of induction failure and cesarean section. Also, the modified Bishop score was found to be important in predicting the success of induction using various agents [7]. Moreover, a retrospective study on women aged 35 or older undergoing prostaglandin-induced labor found that cesarean section and induction failure were associated with many independent risk factors, including advanced maternal age, shorter labor duration, a low Bishop score at induction, low parity, and high newborn weight. Higher parity correlated with a higher rate of vaginal delivery [8]. Additionally, a study from the University Hospital' Le Scotte' of Siena, Italy, emphasized a higher risk of cesarean section linked to maternal age over 35, nulliparity, and a low Bishop score [8]. Regarding Saudi Arabia, a study conducted at King Faisal Military Hospital compared prostaglandin E2 induction to spontaneous labor in grand multiparous patients, showing higher cesarean rates and more need for Syntocinon augmentation in the induced group [9]. In a similar trial at King Abdulaziz University Hospital involving 202 grand multiparous women found significantly higher cesarean rates in those induced with vaginal prostaglandin E2 compared to those with spontaneous labor [10]. Furthermore, factors influencing the success of induction of labor (IOL) were studied at King Khalid University Hospital in 564 women from April 2010 to March 2011. Nulliparity and a high maternal BMI were found to be maternal factors associated with an increased risk of cesarean section during IOL [11]. Extensive research is needed for a better understanding. Therefore, this research paper aims to evaluate the indications and factors associated with successful induction of labor and maternal and neonatal outcomes.

## 2. Methods

A hospital-based retrospective chart review study was carried out in the National Guard Hospital in Riyadh, Saudi Arabia, from January to April 2023. Data were collected using a purposive convenience sampling technique from the induction of labor medical records. All pregnant women who underwent induction of labor at the National Guard Hospital during the specified timeframe were included in the study. A total of 604 pregnant women of all gestational ages with different ethnicities, age groups, obstetric history, and medical indications for labor induction were enrolled in the study. From the initial group, a number of 73 pregnant women were excluded due to contraindications to labor induction, incomplete medical records, multiple pregnancies, or not undergoing labor induction. The collected data, including demographic information, obstetric history, labor induction methods, and labor outcomes, were entered into an Excel spreadsheet and exported to the SPSS version 26 statistical package for comprehensive analysis.

### 2.1. Statistical Analysis

The data were analyzed using the software program Statistical Packages for Software Sciences (SPSS) version 26 (Armonk, New York, IBM Corporation, USA). Descriptive statistics were presented as numbers and percentages (%) for all categorical variables. Univariate analysis was performed to determine the factors that influence the type of delivery. Significant results were placed in multivariate regression analysis to determine the significant independent predictors of cesarean delivery, with corresponding odds ratios and 95% confidence intervals. Values were considered significant with a *p* value of less than 0.05.

## 3. Results

This study analyzed 531 pregnant women. As seen in Table 1, 52.7% were aged 30 years or younger. More than half (55.7%) were considered obese. Nearly two-thirds (64.4%) had a frequency of gravidity of 3 or less, and 42.6% were primiparous. Women who had abortions constitute 32.4%. Late-term women constitute 55%. The most common type of IOL intervention was Prostin (63.5%), wherein 73.3% received at least 1 to 2 doses. Normal delivery was prevalent (62%). 52.5% had a baby birth of more than 3 kg, and 50.3% delivered with a baby boy. Most women were in reassurance levels based on APGAR scores in 1-minute (95.1%) and 5-minute marks (98.6%). In addition, 85.7% were admitted to SCBU indicating normal newborn outcomes.

**Figure 1:**
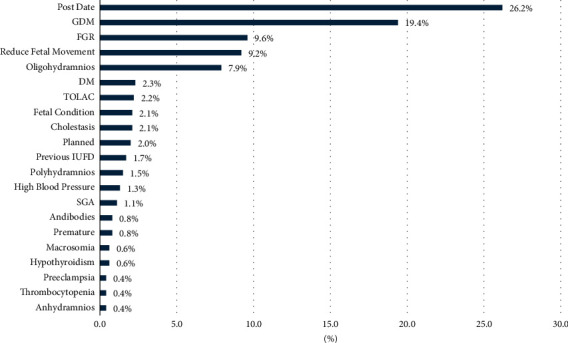
Indication of IOL. GDM = gestational diabetes mellitus, FGR = fetal growth restriction, DM = diabetes mellitus, TOLAC = trial of labor after cesarean, previous IUFD = intrauterine fetal death, SGA = small for gestational age.

Figure 1 illustrates that the most common indication of IOL was postdated pregnancy (26.2%), followed by GDM (19.4%) and FGR (9.6%) (Figure 1).

**Figure 2:**
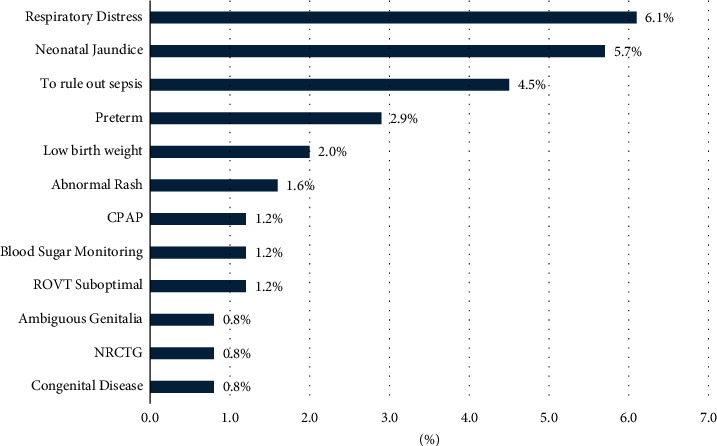
Cause of admission to NICU/ICN. CPAP = continuous positive airway pressure, ROVT suboptimal = right ventricular outflow tract suboptimal, NRCTG = Nonreassuring cardiotocography.

Figure 2 depicts that the most common cause of admission to NICU/ICN was respiratory distress (6.1%), followed by neonatal jaundice (5.7%) and to rule out sepsis (4.5%) (Figure 2).

When measuring the relationship between the type of delivery according to the demographic and clinical characteristics of the pregnant women (Table 2), it was observed that women who had normal deliveries were more likely to be primiparous (*p* < 0.001) and had received Prostin medication for IOL (*p* < 0.001), while women who underwent cesarean delivery were more likely to have lower gravidity (≤3) (*p* < 0.001) and had received Propess medication (*p* < 0.001).

When conducting a multivariate regression analysis (Table 3), it was found that compared to nulliparous women without parity, women who had one parity had an increased chance of having labor success by almost 10.8 times higher (AOR = 10.769; 95% CI = 4.292 – 27.018; *p* < 0.001), while among women with more than one parity, the odds could increase by at least 2.72-fold higher (AOR = 2.716; 95% CI = 1.121–6.578; *p*=0.027). Also, compared to women who did not receive Prostin, women who received Prostin had an increased chance of having labor success by at least 1.93 times higher (AOR = 1.929; 95% CI = 1.133–3.286; *p*=0.016). However, gravidity and Propess have no significant effect on IOL after adjustment to a regression model (*p* > 0.05).

## 4. Discussion

The present study investigated the factors crucial to the success of IOL among pregnant women visiting a tertiary care hospital in Saudi Arabia. This study finds that among 531 women who underwent IOL, 62% were normal deliveries, suggesting IOL success, while 31.1% were cesarean deliveries, which may have been an indication of IOL failure. This result is within the range as documented by several studies within the region, ranging from 56% to 84% of vaginal delivery post-IOL [11–13]. In Brazil, the rate of vaginal deliveries was consistent with previous reports at 72% [14]. Contradicting these reports, a study conducted in Sudan found that among 3834 pregnant women, the prevalence of IOL was only 9%, which is very low compared to studies in developed countries. The rate of delivery using the cesarean method was 24%. The failure rate was also seen to be low (7.2%). However, all failed inductions were delivered using the cesarean method, incrementing by 3.3% of the overall rate of the institution's cesarean deliveries throughout the study duration [15]. In the United States, over the last 30 years, the rates of labor induction markedly increased. Based on data from the National Center for Health Statistics, the rate of labor induction was 9.6% in 1990, 27.1% in 2018, and 31.4% in 2020 [16]. As this trend continues to surge, obstetricians should be well equipped with several methods available for IOL.

Data from our study suggest that increasing parity and Prostin medication were associated with a better IOL success rate. In particular, primiparous and multiparous women had 10.8 and 2.7 threshold increases of successful IOL compared to nulliparous women, while pregnant women who received Prostin had a 1.9-fold higher predicting successful rate. This is almost in agreement with the study of Batinelli et al. [9]. Multiparity was identified as one of the protective factors for vaginal delivery, followed by a high Bishop score, while advanced age (age >35 years) and increased fetal birth weight (>3500 g) were seen to increase the risk of cesarean section. This was supported by the study of Wang et al. [18]. Based on a predictive model, IOL success using high-volume Foley catheterization was associated with parity, age, and height. However, in an investigation done by Al-Shaikh et al., nulliparity and elevated BMI were the influencing factors of the cesarean method, while a higher APGAR score was associated with the success of IOL at delivery [11]. In contrast, Farah et al. revealed that an increased Bishop score, the start of induction to delivery (<12 h), and fetal heart pattern (nonreassuring) were the influential factors of IOL success [19]. Without a doubt, the soft delivery canal of multiparous has a better chance of IOL success since cervical dilation is more easily achieved than in nulliparous women. The selection policy of women for IOL is a key contributor to IOL success, and the circumvention of elective IOL for nonmedical reasons with its recognized associated risks of maternal and perinatal undesirable outcomes and the use of Prostin and Propess to ripen the cervix as previously documented [20].

A critical review published in Canada reported that maternal characteristics of the women were associated with the success of induction, including age, weight, height, BMI, and gestational age [7] This was in agreement with the study done in India [12], wherein women aged between 20 and 25 years, gestational age (>40–42 weeks), and normal BMI were more likely to have a vaginal delivery. In our study, age, BMI level, abortion, gestational age, fetal weight, fetal gender, APGAR scores, and admission to SCBU or NICU have no significant effect between normal and cesarean methods of deliveries (*p* > 0.05).

Moreover, based on univariate analysis, the rates of cesarean delivery were prevalent among women with lower gravidity (≤3) and those who received Propess medication. However, the results of our adjusted model did not yield statistical significance between these factors (*p* > 0.05). Thus, further investigation is required to validate these results. This almost mirrored the paper of Batinelli et al. [9]. Results revealed that increasing Propess time in situ was correlated with the increased interval of induction time to deliver, which ultimately led to an increased risk of cesarean section. This agrees with the study of Bączek et al., with previous cesarean section and higher frequency of deliveries associated with fewer IOLs [21]. In contrast, Favilli et al. disclosed that the Bishop score, parity, and labor time were correlated inversely with higher cesarean rates [8].

It is important to note that postdated pregnancy, gestational diabetes, and FGR were the most common indications of IOL to NICU/ICN. This mirrored the results of studies done in Riyadh, with post-term pregnancies being the most prominent IOL indication. However, in India [12], fetal distress and meconium-stained liquor were identified as the most common indication of cesarean section, while in Ethiopia [13], preeclampsia/eclampsia and prelabor rupture of the fetal membrane were seen as the major reasons for IOL.

## 5. Conclusion

Almost two-thirds of the pregnant women had normal deliveries after IOL. Increasing parity and Prostin administration greatly influence the success of IOL. However, regardless of influence, lower gravidity (≤3) and Propess administration could increase the chance of IOL failure among this population group. Hence, more clinical investigations are needed to be done to improve the success of IOL. Evaluating and surveillance of maternal and fetal status prior to IOL is crucial to its success. Further prospective studies are warranted to determine the cause and effect of the factors influencing IOL success, as identified in this study. The findings of this study could significantly impact local clinical guidelines in Saudi Arabia by providing evidence-based recommendations for local practice. Emphasizing careful patient assessment before IOL, especially regarding gravidity, parity, and induction agents, these findings could optimize protocols, reduce cesarean rates, and improve maternal and neonatal outcomes.

## Figures and Tables

**Table 1:** Demographic and clinical characteristics of the pregnant women (*n* = 531).

Study variables	*N* (%)
*Age group*	
≤30 years	280 (52.7)
>30 years	251 (47.3)

*BMI level*	
Nonobese (<30 kg/m^2^)	235 (44.3)
Obese (≥30 kg/m^2^)	296 (55.7)

*Gravidity*	
≤3	342 (64.4)
>3	189 (35.6)

*Parity*	
Nulliparous	199 (37.5)
Primiparous	106 (20.0)
Multiparous	226 (42.6)

*Abortion*	
Yes	172 (32.4)
No	359 (67.6)

*Gestational weeks*	
≤40 weeks	239 (45.0)
>40 weeks	292 (55.0)

*Type of IOL* ^∗^	
Mechanical	79 (14.9)
Propess	138 (26.0)
Prostin	337 (63.5)

*How many Prostin doses were given (n* *=* *337)*	
1-2	247 (73.3)
3-4	88 (26.1)
≥5	02 (0.60)

*Type of delivery*	
Normal delivery	329 (62.0)
CS delivery	165 (31.1)
Forceps	04 (0.90)
KIWI	32 (06.0)

*Fetal weight*	
≤3 kg	252 (47.5)
>3 kg	279 (52.5)

*Fetal gender*	
Boy	267 (50.3)
Girl	264 (49.7)

*APGAR score 1 min (n* *=* *514)*	
Concerning (0–3)	03 (0.60)
Moderately normal (4–6)	22 (04.3)
Reassuring (7–10)	489 (95.1)

*APGAR score 5 min (n* *=* *514)*	
Concerning (0–3)	01 (0.20)
Moderately normal (4–6)	06 (01.2)
Reassuring (7–10)	507 (98.6)

*Admission to SCBU or NICU*	
Yes, ICN	24 (04.5)
Yes, NICU	51 (09.6)
Yes, SCBU	455 (85.7)
Yes, morgue	01 (0.20)

^∗^Some patients have multiple types of IOL.

**Table 2:** Relationship between the type of delivery among the demographic and clinical characteristics of the pregnant women (*n* = 494)^∗^.

Factor	Type of delivery		*P* value^§^
	Normal *N* (%) (*n* = 329)	Cesarean *N* (%) (*n* = 165)	
*Age group*			
≤30 years	176 (53.5)	82 (49.7)	0.425
>30 years	153 (46.5)	83 (50.3)	

*BMI level*			
Nonobese	139 (42.2)	78 (47.3)	0.289
Obese	190 (57.8)	87 (52.7)	

*Gravidity*			
≤3	189 (57.4)	124 (75.2)	<0.001^∗∗^
>3	140 (42.6)	41 (24.8)	

*Parity*			
Nulliparous	79 (24.0)	96 (58.2)	<0.001^∗∗^
Primiparous	75 (22.8)	27 (16.4)	
Multiparous	175 (53.2)	42 (25.5)	

*Abortion*			
Yes	108 (32.8)	53 (32.1)	0.875
No	221 (67.2)	112 (67.9)	

*Gestational weeks*			
≤40 weeks	154 (46.8)	70 (42.4)	0.356
>40 weeks	175 (53.2)	95 (57.6)	

*Type of IOL* ^∗^			
Mechanical	44 (13.4)	29 (17.6)	0.215
Propess	60 (18.2)	59 (35.8)	<0.001^∗∗^
Prostin	237 (72.0)	88 (53.3)	<0.001^∗∗^

*Fetal weight*			
≤3 kg	149 (45.3)	81 (49.1)	0.424
>3 kg	180 (54.7)	84 (50.9)	

*Fetal gender*			
Boy	155 (47.1)	88 (53.3)	0.192
Girl	174 (52.9)	77 (46.7)	

*APGAR score 1 min (n* *=* *477)*			
0–6	13 (04.1)	11 (07.0)	0.175
7–10	306 (95.9)	147 (93.0)	

*APGAR score 5 min (n* *=* *477)*			
0–6	05 (01.6)	02 (01.3)	0.797
7–10	314 (98.4)	156 (98.7)	

*Admission to SCBU or NICU*			
Yes, ICN	14 (04.3)	09 (05.5)	0.790
Yes, NICU	29 (08.8)	16 (09.7)	
Yes, SCBU	285 (86.9)	140 (84.8)	

^∗^Some patients have multiple types of IOL. ^§^*P* value has been calculated using the chi-square test. ^∗∗^Significant at the *p* < 0.05 level.

**Table 3:** Multivariate regression analysis to determine the factor that influences labor success (*n* = 494)^∗^.

Factor	AOR	95% CI	*P* value
*Gravidity*			
≤3	Ref		
>3	0.475	0.205–1.102	0.083

*Parity*			
Nulliparous	Ref		
Primiparous	10.769	4.292–27.018	<0.001^∗∗^
Multiparous	2.716	1.121–6.578	0.027^∗∗^

*Propess*			
No	Ref		
Yes	1.891	0.954–3.750	0.068

*Prostin*			
No	Ref		
Yes	1.929	1.133–3.286	0.016^∗∗^

AOR: adjusted odds ratio; CI: confidence interval. ^∗^Other types of delivery were excluded from the analysis. ^∗∗^Significant at the *p* < 0.05 level.

## Data Availability

The data that support the findings of this study are available on request from the corresponding author. The data are not publicly available due to privacy or ethical restrictions.
